# Family systemic psychosocial support for at-risk adolescents in Lebanon: study protocol for a multi-site randomised controlled trial

**DOI:** 10.1186/s13063-022-06284-y

**Published:** 2022-04-18

**Authors:** Felicity L. Brown, Tania Bosqui, Joseph Elias, Sally Farah, Anas Mayya, Diana Abo Nakkoul, Bryony Walsh, Sarah Chreif, Ahmad Einein, Bassel Meksassi, Roula Abi Saad, Hady Naal, Maliki E. Ghossainy, Michael Donnelly, Theresa S. Betancourt, Alan Carr, Eve Puffer, Rabih El Chammay, Mark J. D. Jordans

**Affiliations:** 1 Research and Development Department, War Child Holland, Amsterdam, The Netherlands; 2 Amsterdam Institute of Social Science Research, University of Amsterdam, Amsterdam, The Netherlands; 3 Department of Psychology, American University of Beirut, Beirut, Lebanon; 4 Trinity Centre for Global Health, Trinity College Dublin, Dublin, Republic of Ireland; 5 War Child Lebanon, Beirut, Lebanon; 6 Terre des Hommes Italy, Beirut, Lebanon; 7 Danish Refugee Council, Beirut, Lebanon; 8 Child Protection Department, United Nations Children’s fund (UNICEF), Lebanon, Beirut, Lebanon; 9 Global Health Institute, American University of Beirut, Beirut, Lebanon; 10 Wheelock College of Education and Human Development, Boston University, Boston, MA USA; 11 Centre for Public Health, School of Medicine, Queen’s University Belfast, Belfast, Northern Ireland; 12 Boston College School of Social Work, Chestnut Hill, MA USA; 13 School of Psychology, University College Dublin, Belfield, Dublin, Republic of Ireland; 14 Department of Psychology & Neuroscience, Duke Global Health Institute, Duke University, Durham, NC USA; 15 Ministry of Public Health, Beirut, Lebanon; 16 Department of Psychiatry, Faculty of Medicine, Saint Joseph University, Beirut, Lebanon

**Keywords:** Psychological intervention, Family therapy, Mental health and psychosocial support, Randomised controlled trial, Study protocol, Low- and middle-income countries (LMICs), Lebanon, Refugees, Armed conflict, Adolescents

## Abstract

**Background:**

Adolescents growing up in communities characterised by adversity face multiple risk factors for poor mental health and wellbeing. There is currently a scarcity of research on effective approaches for preventing and treating psychological distress in this population, particularly in humanitarian settings. The powerful impact of the home environment and family support is well known; however, approaches targeting the family are seldom developed or evaluated in such settings. We developed a brief family systemic psychosocial support intervention to be delivered through existing child protection systems with non-specialist facilitators. This paper outlines the study protocol for a randomised controlled trial of the intervention in Lebanon.

**Methods:**

We will conduct a single-blind hybrid effectiveness-implementation multi-site RCT comparing the locally developed systemic family intervention to a waitlist control group for families residing in vulnerable regions in North Lebanon and Beqaa governorates (including families of Syrian, Palestinian, and Lebanese backgrounds). Outcomes on a range of family, adolescent, and caregiver measures will be assessed at baseline (T0) and post-intervention (T1), and at a 3-month follow-up for the treatment arm (T2). Families will be eligible for the trial if they are identified by implementing organisations as being medium-to-high risk for child protection concerns and have one or more adolescent aged 12–17 who demonstrates significant psychological distress on a self-report brief screening tool. Families will be randomly assigned to a treatment or a waitlist control condition. Families in the waitlist condition will receive a group version of the programme after completion of the study, to allow us to assess feasibility, acceptability, and preliminary indications of intervention effects of this modality. The primary outcome is reduction in overall adolescent-reported psychological distress over time, with post-intervention (T1) as the primary endpoint. Secondary adolescent-reported outcomes include family functioning, psychosocial wellbeing, and emotional regulation difficulties. Secondary caregiver-reported outcomes include parenting style, family functioning, psychological distress, and emotional regulation difficulties.

**Discussion:**

This trial will provide the first assessment of the effectiveness of the family systemic psychosocial support intervention for use in Lebanon, with important implications for the use of systemic, low-cost, non-specialist interventions for this age range.

**Trial registration:**

Local registry:

National Mental Health Program, Ministry of Public Health, Lebanese Republic. Registered on 19 October 2021

Lebanese Clinical Trial Registry LBCTR2021104870. Registered on 13 October 2021

Global registry: ISRCTN ISRCTN13751677. Registered on 1 November 2021

## Background

Adolescence is a critical period for shaping future mental health and wellbeing, yet adolescents living in adversity, particularly those growing up in humanitarian emergencies, face myriad risk factors. These include increased family and community violence, poverty and associated daily stressors, limited educational opportunities, other protection concerns, and lack of access to adequate services. In addition to this, access to quality mental health services is often severely constrained due to multiple factors including limited number of mental health professionals, lack of availability of affordable care, and stigma.

Recently, research and practice have turned attention to innovative task-shifting approaches, whereby non-specialists are trained and supervised to deliver mental health and psychosocial support interventions, and provide referrals to specialist services for the smaller number of individuals requiring them. Evidence from an increasing number of trials for psychological interventions with adults [1] and children and adolescents [2] has shown that this can be a safe and effective way to increase accessibility of care.

Despite knowledge of the importance of nurturing environments for promoting good mental health and wellbeing for children into adulthood [3], and of the effectiveness of family therapy [4], limited programmes have been developed and tested to support families in low resource and humanitarian settings. A systematic review of the literature in 2017 [5] found only 32 parenting and family interventions evaluated in low- and middle-income countries. We updated this review in 2020 (Bosqui T, Mayya A, Farah S, Shaito Z, Betancourt T, Carr A, Donnolly M, Pedersen G, Brown, F: A systematic review of the evidence for the effectiveness and implementation of family and parenting interventions in lower and middle-income countries for child and adolescent mental health, in preparation) and found 43 new studies; however, very few focus on the family as a whole, very few are evaluated in conflict-affected settings and/or in the middle-east region, and most are preventive rather than supporting families already identified as at-risk or in distress. No family-focused interventions have been trialled to date in Lebanon.

Through a series of collaborative workshops with non-governmental organisation, United Nations, academic, and governmental partners, alongside consultation with local and international experts and a Community Advisory Board (CAB) from the target communities, we developed a systemic family psychosocial support intervention designed to be delivered to families of at-risk adolescents through the existing child protection system in Lebanon. The intervention comprises six, 90-min family sessions, plus 30-min parent-only sessions each week, and a booster session 1 month later. The sessions target key family skills (described in methods) identified to be important through our extensive qualitative interviews with families, parents, adolescents, and experienced psychosocial support facilitators (Farah S, Brown F, Mayya A, Shaito S, Elias J, Betancourt T, Carr A, Donnolly M, Bosqui, T: Qualitative exploration of family functioning and mental health for at-risk adolescents in Lebanon, in preparation), and through analysing key intervention components and implementation models found in effective family interventions (Brown FL, Bosqui T, Elias J, Farah S, Mayya A, Betancourt T, Carr A, Donnolly M, Abou Naccoul D, Walsh B, Abi Saad R, Naal H, Shaito Z, Ghossainy M, Jordans MJD: development of the ‘Sawa Aqwa (Stronger Together)’ Family Focused Psychosocial Support Program for at-risk adolescents in Lebanon, in preparation). Key features of the intervention (referred to throughout as ‘family intervention’) include that it (i) involves the whole family; (ii) is transdiagnostic in nature, meaning that it targets families experiencing psychological distress broadly; (iii) is designed specifically for families living in communities characterised by adversity and acknowledges their lived reality; and (iv) can be safely delivered by non-specialists.

This paper presents the study protocol for the randomised controlled trial (RCT) being conducted in Lebanon to determine the effectiveness of the family intervention.

## Methods

### Design

**Fig. 1:**
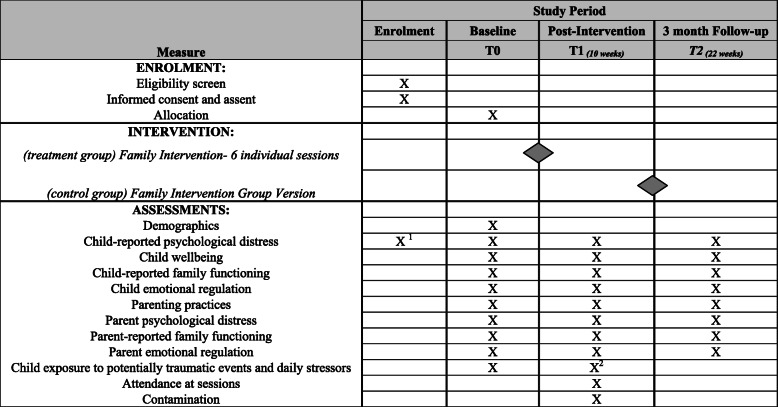
Standard Protocol Items Recommendations for Interventional Trials (SPIRIT): schedule of enrolment, interventions, and assessments. ^1^17-item screener. ^2^Daily stressors only

The study is a single-blind type I hybrid effectiveness-implementation multi-site superiority RCT comparing the locally developed family intervention to a waitlist control group for families residing in vulnerable regions in North Lebanon and Beqaa governorates (including families of Syrian, Palestinian, and Lebanese backgrounds). Outcomes on family, adolescent, and caregiver measures will be assessed at baseline (T0) and post-intervention (T1), and at a 3-month follow-up for the treatment arm (T2). The primary outcome point is set as T1. The Standard Protocol Items: Recommendations for Interventional Trials (SPIRIT) is outlined in Fig. 1. A waitlist comparison condition was chosen in order to enable us to deliver a group-format version of the intervention with the control arm after T1 assessments are complete, to gather implementation data and experiences of this format.

### Aims and hypotheses

The primary aim of the RCT is to assess the effectiveness of the family intervention on adolescent-reported symptoms of psychological distress at post-intervention. The secondary aim is to assess the effectiveness of the intervention using other measures of family functioning, parenting, and adolescent and caregiver mental health and wellbeing from pre- to post-intervention, and retention of effects in the treatment group at a 3-month follow-up timepoint. An additional aim is to explore possible treatment moderators (e.g. age, gender, past traumatic exposures, level of adversity, income). We will also explore whether treatment effects for adolescents and caregivers are mediated by improvements in (adolescent- and caregiver-reported) family functioning and (adolescent- and caregiver-reported) emotional regulation, and whether treatment effects for adolescents are mediated by improvements in caregiver outcomes.

Our hypotheses are:

i) Adolescents in families assigned to receive the family intervention will show significantly greater improvements in primary and secondary outcome measures compared to the waitlist group, at post-intervention.

ii) Caregivers in families assigned to receive the family intervention will show significantly greater improvements on caregiver-reported secondary outcome measures compared to the waitlist group, at post-intervention.

iii) These improvements for adolescents and caregivers will be maintained at the 3-month follow-up time point for the treatment condition.

iv) In the waitlist families who receive the intervention in a group format, we predict that there will be significant within-group improvements on outcome measures.

### Setting

The study will be implemented by three implementing organisations (i.e. three sites) operating in two governorates of Lebanon—primarily in urban areas in North Lebanon (War Child Lebanon [WC], Danish Refugee Council Lebanon [DRC]) and in informal tented settlements in agricultural areas in north Beqaa (Terre Des Hommes Italy [TDH], Lebanon). Each implementing organisation has existing child protection programming in these areas.

Lebanon is a middle-income country with a recent history of internal and external armed conflict, facing an unprecedented economic crisis, exacerbated by the COVID-19 pandemic and the Beirut port explosions which caused mass devastation in the capital city. Lebanon also hosts the highest number of refugees per capita—with a current estimate of 1.5 million Syrian refugees, in addition to large numbers of Palestinian refugees, in a total national population of 5.9 million [6]. The current political paralysis, economic crisis, and this rapid and large increase in the population over a short period of time have meant that the ability to meet educational, health, financial, and mental health needs of the population is severely limited. Since the onset of the devaluation of the Lebanese Lira, over three quarters of the population are living in poverty with frequent shortages of fuel, essential medicines, and electricity [7]. The situation in Lebanon has taken its toll on the population, including soaring rates of mental ill health, suicide, and child protection violations [8]. Prior to the crisis, estimates indicated there were only 1.26 psychiatrists and 3.42 psychologists per 100,000 population, with 97% working in the private sector, making mental health care inaccessible to the most vulnerable [9].

### Participants

Families will be included if they meet the following inclusion criteria: (i) have an adolescent aged 12–17 years (male and female), (ii) are identified as medium to high risk for child protection concerns by the implementing organisation, (iii) give assent and parental/legal guardian consent to take part in trial procedures, and (iv) at least one adolescent scores above the cut-off on the Pediatric Symptom Checklist (PSC-17) for general psychological distress at screening. Families of any nationality and background will be eligible (with an expected focus on Lebanese, Syrian, and Palestinian families), as well as families of any composition (including single or dual headed households), provided that an adult legal guardian is present. Child protection risk status will be established as part of usual screening and outreach processes for focused psychosocial support by staff in implementing organisations, who will identify and refer potential participants. Medium-to-high risk is defined for this study as being ‘vulnerable to a protection risk but not high with imminent risk’.

Participants will be excluded if they meet any of the following criteria: (i) imminent risk requiring immediate case management at the time of recruitment, or if they have severe psychiatric disturbance or risks requiring specialist mental health services (assessed by implementing organisations as part of usual routine assessment and referral systems); (ii) unaccompanied, separated, or married, due to challenges with the legal consent of guardians; and (iii) engaged in case management at the time of outreach, as those in case management are likely to be receiving sufficient services. If the need for case management arises during the intervention, then families can be referred as usual and remain in the study.

### Sample size

The sample size calculation was based on a two-group comparison of primary outcome at the post-intervention time point. Assuming a 5% two-tailed significance test, 80% power, it is estimated that data from 270 participants would need to be available at the post-intervention time point in order to detect an effect size of .25. Allowing for 30% attrition, this would correspond to an overall sample size of 351 required at enrolment. A recent individual patient data meta-analysis [10] of psychosocial support interventions for children in low-resource humanitarian settings found an effect size for post-traumatic stress symptoms of 0.33, and functional impairment of 0.29, with no significant effect for depression and anxiety. While our primary outcome is a measure of broad child psychological symptoms, we felt these estimates were most applicable to our study context. Given the novel family systems approach we are employing, we aimed for a conservative minimal detectable effect of 0.25.

### Recruitment

Recruitment will be conducted through implementing organisations (TDH, WC, DRC) using various outreach strategies including: i) door to door outreach conducted by community outreach teams, ii) referral from existing programmes in the organisation; iii) referral from community-based organisations, and iv) word of mouth resulting in families self-referring and contacting the implementing organisation. Recruitment will be guided by a standard script. At the point of outreach implementing organisations will assess for level of risk using standard procedure, identifying families at medium-high risk usually eligible for focused-psychosocial support programmes, with an adolescent aged 12–17 and consenting to take part. Families meeting these preliminary inclusion criteria will be registered and asked permission for their details to be shared with the research team at the American University of Beirut (AUB).

### Informed consent and assent

Registered participants will be contacted by a research assistant at AUB, and informed consent and assent will be gained over the phone from caregivers and adolescents respectively. The documents will be sent over the phone and read through with oral consent and assent taken. An independent witness (another family member, friend, or neighbour) will be requested to be present during the consent process. The research assistant will sign the form and note the oral consent, and a copy of this will be sent to the family over the phone (via WhatsApp or email). For families who are unable to receive the documents electronically, hard copies will be delivered to their home.

### Screening

After obtaining informed consent and assent, the research assistant will record family demographic data and administer the screening interview over the telephone with each adolescent aged between 12 and 17 years old. Localised guidance [11] to assess participants over the phone and in cramped living spaces will be followed to maximise the privacy of respondents during assessments and to respond to distress or discomfort.

The screening interview with adolescents will consist of the PSC-17 (adolescent report), a brief version of the 35-item PSC measuring symptoms of internalising and externalising difficulties in children and adolescents [12]. The tool has been validated with young adolescents in Lebanon, using semi-structured interviews with psychiatrists as the gold standard [13], and a cut-off of 12 or more is indicative of a need for psychosocial treatment. The family will be eligible to take part if at least one adolescent screens positive on the PSC-17. Where more than one adolescent screens positive, the adolescent scoring the highest will be selected as the index adolescent, and only this adolescent will complete outcome measures at T0 and remaining time points. In the unlikely event of multiple adolescents in the same family scoring the same total score, the oldest will be selected as the index adolescent.

### Randomisation

Randomisation will occur after T0 assessments are completed and will be stratified by site. The unit of randomisation will be families, and they will be randomly allocated to intervention or waitlist with a 1:1 ratio. The randomisation sequence will be computer-generated by a statistician located outside Lebanon, and independent of the study. It will be generated using Research Randomizer (randomizer.org [14]), a computer-based pseudo-random number generator using the “Math.random” method within the JavaScript programming language as the core methods for generating its random numbers. Families are randomised into the intervention group (see details in the “Intervention” section), or a waitlist condition, to receive the programme after post-intervention assessments (T1). They will receive the programme in a group format to assess the feasibility and acceptability of different delivery formats.

To ensure approximately equal randomisation in case smaller numbers of families per organisation are attained than planned, blocking will be used with block sizes unknown to the research team. An unblinded study coordinator will send participant lists (in order of baseline assessment completion date and time) to the independent statistician on a regular basis during the study enrolment period. The independent statistician will then match the randomly generated allocation sequence to the participant list and send this back to the unblinded study coordinator, who will then share participant allocations with implementing teams.

### Outcome measures

We will aim to commence intervention sessions no more than 4 weeks after T0 assessments, and we will aim to conduct T1 assessments within 4 weeks of the 6th session (i.e. approximately 10 weeks after T0); T2 assessments will be scheduled at 12 weeks following T1 (i.e. approximately 19 weeks after T0). Participants will receive 25,000 LL (valued at around $1 using market rates at the time of data collection, due to devaluation of the currency, however providing ample phone data for assessments) per family per assessment to reimburse them for data usage and their time. In case participants do not attend a scheduled assessment, three attempts will be made to contact them in the same week, and once the following week, to schedule a new appointment. This will be done via phone calls, or contacting alternative contacts provided.

The majority of measures have been used in Lebanon previously by the study team in different studies [15, 16]. Two measures that had not been used in Lebanon previously—the Systemic Clinical Outcome and Routine Evaluation (SCORE) Index of Family Functioning [17] and the Difficulties in Emotion Regulation Scale-Short Form (DERS-SF [18])—were translated into simple, non-formal Arabic that can be understood by participants in the region (i.e. Syrians, Lebanese, Palestinians) following recommended procedures for cross-cultural research [19]. Steps involved were as follows: forward translation to Arabic by four team members; independent back translation to English by two people; workshops with English-speaking and bilingual team members to review the translations and ensure they retained the original English meaning; cognitive testing with CAB members to assess comprehensibility, completeness, relevance, and acceptability; review workshops to adjust as needed; and pilot testing with target populations.

All instruments will be delivered via individual telephone interviews by trained research assistants, using the KoBo electronic data collection software on tablets. Assessments will be conducted with the index adolescent, and all caregivers where possible. Prior to taking part in the study, assessors will receive training on the assessments with adolescents and parents, phone delivery, sensitive interviewing, managing distress and risk management, research ethics, gaining informed consent and assent, study procedures and record keeping (using RedCap and KoBo), provision of compensation, risks for bias in collecting quantitative data, adverse events reporting procedures, and data management. Assessors will also role-play the full process from scheduling to assessment and data collection, in Arabic, with multiple opportunities for practice role-plays before taking on families. Ongoing monitoring of assessors’ competency and consistency will be conducted through regular supervision by the PIs.

#### Primary outcome

The primary outcome is psychological distress, as assessed by the Pediatric Symptom Checklist 35 (PSC-35) youth-report [20]. The PSC-35 consists of 35 symptoms (including internalising, externalising, somatic, social, and academic difficulties), rated for their frequency on a 3-point scale ranging from 0 (never) to 2 (often). The total PSC-35 score is obtained by summing scores of individual items and ranges from 0 to 70. In a validation study in Lebanon, the measure showed high internal consistency (*σ* = .80), convergent validity, test-retest reliability, and concurrent validity with psychiatrist clinical assessments [13]. The 17 items of the PSC-17 are identical to those in the PSC-35, and therefore at baseline, screening scores for adolescents on these items will be used, and only the remaining 18 items assessed. We will ensure that there is a maximum of 2 weeks between screening and baseline assessments.

#### Secondary outcomes

##### Adolescent-reported outcomes

Adolescent-reported wellbeing will be measured with WHO-5 Wellbeing Index [21, 22]. The WHO-5 consists of 5 questions asking participants to rate their answers over the last week on a scale of from 0 (none of the time) to 5 (all of the time). Total scores range from 0 to 25, with lower scores indicating lower wellbeing. The WHO-5 Arabic version has been tested within Syrian refugee populations in Lebanon with acceptable psychometric properties (Pluess M, McEwen F, Moghames P, Chéhadé N, Bosqui T, Hijazi A, Murray L, Skavenski S, Bolton P, Weierstall R, Legoff S, Hassan Z: Development, piloting, and evaluation of telephone delivered Common Elements Treatment Approach (t-CETA) for Syrian refugee children in Lebanon, in preparation).

Adolescent-reported emotional regulation will be measured with the DERS-SF. The DERS-SF consists of 18-items, with participants rating their answers in the past week on a scale from 1 (almost never) to 5 (almost always). Total scores range from 18 to 90, with higher scores indicating more difficulty in emotional regulation. As described in detail above, an Arabic version was developed for the study with permissions from the authors.

Adolescent-reported family functioning will be measured with the SCORE. The 15-tem measure asks participants to rate whether the item describes their family on a scale from 1 (not at all) to 5 (very well) in the past week. Total scores range from 15 to 75, with a lower score indicating higher family functioning. An Arabic version was developed for the study with permission from the authors.

##### Caregiver-reported outcomes

Caregiver psychological distress will be measured using the Kessler Psychological Distress Scale (K6 [23]). The K6 consists of six questions asking about mental health in the previous week, which are scored on a scale from 1 (all of the time) to 5 (none of the time). Total scores range from 6 to 30, calculated by summing individual items. Lower scores indicate higher levels of psychological distress.

Caregiver-reported parenting skills will be measured with the War Child Parenting Scale. The 26-item scale developed in Lebanon as part of a parenting intervention trial (Chen A, Koppenol-Gonzalez GV, Bakolis I, Arnous M, Tossyeh F, Al-Ogaily D, Jordans MJD, Miller KE: Dimensions of Parenting Scale: a culturally grounded measure of parenting in adversity, in preparation) asks parents to rate their parenting practices over the last week on a scale from 1 (rarely) to 3 (often). Total scores range from 26 to 78, with higher scores indicating healthier parenting practices.

Caregiver-reported emotional regulation challenges will be measured with the same 18-item Difficulties in Emotion Regulation Scale-Short Form (DERS-SF) as for adolescents.

Caregiver-reported family functioning will be measured with the 15-item Systemic Clinical Outcome and Routine Evaluation (SCORE) Index of Family Functioning as for adolescents.

##### Other measures

In order to measure exposure to traumatic events and daily stressors in adolescents as a demographic characteristic and possible moderator of treatment effects, we developed a 36-item inventory to be asked to caregivers. The list was adapted from a traumatic events checklist used in a recent RCT in Lebanon [15], with trauma items being adjusted and daily stressors being added based on formative research for this trial (Farah S, Brown F, Mayya A, Shaito S, Elias J, Betancourt T, Carr A, Donnolly M, Bosqui, T: Qualitative exploration of family functioning and mental health for at-risk adolescents in Lebanon, in preparation). Each item is scored as ‘yes’ or ‘no’ for occurrence. At T0, we will assess whether these events occurred ever, or in the last month. At T1, we will assess whether daily stressors were present in the last month.

### Intervention

The intervention is a six-session family intervention to be delivered by non-specialist facilitators. Family members will attend weekly two-hour long sessions together, and the programme will be delivered by one facilitator with one family. The first 1.5 h is delivered to the family unit. The final 0.5 h consists of a brief session for the caregivers only. After the 6th session, a booster session of 2 h will be provided after approximately 1 month. The programme was developed by the study team and partners through formative research (Brown FL, Bosqui T, Elias J, Farah S, Mayya A, Betancourt T, Carr A, Donnolly M, Abou Naccoul D, Walsh B, Abi Saad R, Naal H, Shaito Z, Ghossainy M, Jordans MJD: development of the ‘Sawa Aqwa (Stronger Together)’ Family Focused Psychosocial Support Program for at-risk adolescents in Lebanon, in preparation) and covers these main skills: identifying family values, goals, challenges, and strengths; emotional regulation; communication; problem management; managing disagreements; and positive parenting for adolescents. The programme is intended to be delivered face to face in a home visit or in a community centre; however, if safety concerns or restrictions related to COVID-19 prevent this, online modalities will be employed.

Families in the control group will receive the family intervention after T1 assessment. In order to further test the implementation model of the family intervention, the control group will receive a multi-family version of the intervention. Families will be assessed again after the intervention and will also be interviewed to identify implementation and acceptability differences between the versions. Other implementation data, like attendance, fidelity, and dropout, will be collected for both versions of the intervention.

#### Other interventions

Participants will not be prevented from taking part in other interventions during the trial period. There will be no special criteria for discontinuing or modifying allocated interventions. We will ask participants at T1 about other services accessed during the study period.

### Facilitator selection, training, and supervision

Intervention facilitators will be non-specialist providers of any gender recruited through implementing organisations using standard criteria and processes for focused psychosocial support facilitators (harmonised between organisations for this study). They will receive 9 days of training in basic counselling skills and competencies; competencies for working with adolescents, caregivers, and the family system; delivering the content of the intervention; and self-care. At the end of the training, all facilitators will undergo an assessment of competencies in order to be eligible to implement the intervention. Weekly supervision will be provided by a local supervisor (JE), who has Masters level psychology training and has received prior training and mentorship in supervisory techniques. This will  ensure protocol adherence and support for facilitators. In addition, the supervisor will receive regular master supervision with the principal investigators (FB, TB) and a local clinical psychologist, to monitor quality and fidelity of implementation, give input on challenging cases, and provide support in supervision.

### Fidelity

To evaluate treatment fidelity, facilitators will complete a session checklist at each session to indicate which components were completed, which family members attended, and whether any rescheduling was needed. A sample of approximately 5% of sessions will be observed by a trained supervisor, who will complete a structured observation form, developed for this intervention, to score which elements of the programme have been carried out by the facilitator and to what quality.

The competency of facilitators will be assessed at the end of training via a 12-item structured rating form, based on the Enhancing Assessment of Common Therapeutic factors [24] rating scale for training and supervision, adapted for working with families. Five of these competency items will also be assessed during each session observation.

### Ethics and trial management

The trial has received local ethical approval from the Institutional Review Board of American University of Beirut (Protocol ID: SBS-2021-0102, 12 October 2021) and will undergo continuing review on an annual basis. On the informed consent form, participants are informed that the study coordinator, or another clinician other than their therapist, is available to them if they are upset by this study, and they are provided with IRB contact details if they wish to raise a concern.

A trial management committee consisting of principal investigators, co-investigators, partner staff, and research coordinator will meet regularly through a series of weekly, fortnightly, and monthly meetings to monitor different aspects of the implementation of study procedures. All adverse events (AEs; e.g. injuries on the way to treatment, increase in distress) and serious adverse events (SAEs; e.g. suicide attempts, serious violence) will be recorded by the research team and reported to a Data Safety Management Board (DSMB), and appropriate response provided. DSMC meetings will be facilitated monthly by a principal investigator, and the board will consist of 3 or more local professionals, external to the study, but with experience in similar research. The PIs are responsible for reporting (S)AEs to the board, and also to relevant ethics committees. The chair or a nominated person from the DSMB will review SAEs within 48 h and the DSMB will review all AEs once a month and where necessary to determine any appropriate action in respect of ongoing trial conduct. If necessary, appropriate action will be taken with respect to individual participants, or conduct of the trial (such as referral to specialised care, installing extra assessment points for monitoring participants, or discontinuation). No interim analyses are planned. The local study coordinator is responsible for ensuring timely follow-up of any (S)AEs and will inform the participants and DSMB if any data indicate that the disadvantages of participation may be significantly greater than expected.

In the case of protocol amendments, the funder will be notified first, followed by requesting amendments from the IRB. The PIs will notify all partners and share updated protocols and standardised operating procedures, and trial registries will be updated. Any deviations from the protocol will be fully documented in a protocol breach log.

### Blinding

Participants and implementation staff will not be blind to participant allocation. The research assistant team completing T1 assessments will remain blind to the intervention allocation of families throughout the trial and will operate independently from the intervention facilitators. All staff have been trained and supervised in the importance of maintaining blinding, and at no time will intentional unblinding of the research assistants be required. Prior to conducting each T1 assessment, instructions will be given by research assistants to all participants about the importance of not revealing their allocation. If the allocation is revealed, research assistants will inform the research coordinator immediately and where possible another research assistant will complete the assessment with that participant and the remaining family members. At the end of each T1 assessment, research assistants will guess which treatment the participant received—if blinding was maintained, this should be no better than chance.

### Contamination

In order to assess the extent of contamination between arms, at T1 participants from the treatment arm will be asked several structured questions about the extent to which they shared information and materials about the treatment received with others in the community, and participants from the waitlist arm will be asked about whether they have heard about the other treatment and materials from others. This information will be used descriptively to determine contamination.

### Analysis

All analyses will be detailed in a statistical analysis plan, which will be signed before unmasking the study data set. Data will be downloaded from the Kobo data collection software and imported into statistical analysis software for data management and analysis. Details of data security and storage can be found in ethical protocols, which are available on request. Data will be co-owned by the PIs’ organisations, data sharing agreements will be signed by all organisations and co-investigators, and data will be made available on request. All study team members meeting criteria for authorship will be invited to co-author papers presenting the findings.

To determine comparability between the conditions at baseline, multiple planned comparisons will be conducted for continuous variables and chi-squared tests for categorical ones; significance testing will be adjusted for multiple comparisons.

An intent-to-treat analysis approach will be used to test for statistically significant differences over time on primary and secondary outcomes between the two conditions. Hierarchical linear modelling (HLM) will be conducted for each continuous outcome, using a group × time interaction analysis. HLM allows the number of observations to vary between participants and effectively handles missing data. Time (linear and quadratic), treatment condition, and their interaction will be included in the models. Fixed effects parameters will be tested for intervention conditions, and time of assessments at 95 % confidence intervals. Level 1 of the model will represent within-patient change over time on the outcomes of interest, and level 2 of the model will include variables in which the patients are nested to provide better estimates of the within-patient change over time. Covariates will be added and model fit parameters will be used to determine the best fitting models for our data.

Analysis will focus on the primary outcome (PSC-35) and secondary outcomes between treatment and waitlist, with the main outcome point being post-intervention, relative to baseline. Completer analyses will also be conducted using only the data of participants completing the allocated intervention as planned. In addition to the primary analysis, subsequent exploratory analyses will be conducted to consider potential moderators and mediators on outcomes (independent from primary analyses). Across all analyses, two-tailed tests will be reported with *p* < 0.05. In parallel with quantitative analyses, information and observations on implementation will also be analysed to inform the dissemination of the programme.

## Discussion

The family systemic psychosocial support intervention has been developed with the aim of improving mental health and psychosocial outcomes for adolescents living in adversity, through targeting the family system. The module has been locally developed to increase access to relevant, evidence-based, holistic care, with delivery through non-specialist facilitators and requiring minimal resources. It has been designed to be brief and able to be integrated into existing child protection services in Lebanon and other humanitarian settings, including as an adjunctive intervention for existing adolescent-focused programming, or case management services. This is the first trial of a family-focused intervention in Lebanon and will add to the limited evidence-based for family interventions in LMICs more broadly. Findings will be disseminated to academics, practitioners, policy makers, and the communities locally and globally. If effectiveness is demonstrated, it may be scaled up in Lebanon through partners and sector working groups, and adapted and scaled out to adolescents experiencing adversity in other settings.

## Trial status

Recruitment commenced in November 2021 and is expected to continue until April 2022.

*Date and version identifier:* Version 2, 31 March 2022

## Data Availability

Sharing of data and materials, including deidentified individual clinical trial participant-level data, will be considered on request to Tania Bosqui, American University of Beirut, and Felicity Brown, War Child Holland.

## Abbreviations

AEs: Adverse events
AUB: American University of Beirut
CAB: Community Advisory Board
DERS-SF: Difficulties in Emotion Regulation Scale-Short Form
DRC: Danish Refugee Council Lebanon
DSMB: Data Safety Management Board
HLM: Hierarchical Linear Modelling
K6: Kessler Psychological Distress Scale
LMICs: Low- and middle-income countries
PI: Principal investigator
PSC-17: Pediatric Symptom Checklist 17
PSC-35: Pediatric Symptom Checklist 35
RCT: Randomised controlled trial
SAEs: Serious adverse events
SCORE: Systemic Clinical Outcome and Routine Evaluation Index of Family Functioning
SPIRIT: The Standard Protocol Items: Recommendations for Interventional Trials
TDH: Terre Des Hommes Italy, Lebanon
WC: War Child Lebanon
WHO-5: WHO-5 Wellbeing Index
